# Exposure to *Leishmania braziliensis* Triggers Neutrophil Activation and Apoptosis

**DOI:** 10.1371/journal.pntd.0003601

**Published:** 2015-03-10

**Authors:** Sarah A. C. Falcão, Tiffany Weinkopff, Benjamin P. Hurrell, Fabiana S. Celes, Rebecca P. Curvelo, Deboraci B. Prates, Aldina Barral, Valeria M. Borges, Fabienne Tacchini-Cottier, Camila I. de Oliveira

**Affiliations:** 1 Centro de Pesquisas Gonçalo Moniz, FIOCRUZ, Salvador, Bahia, Brazil; 2 Department of Biochemistry, and WHO-Immunology Research and Training Center, University of Lausanne, Epalinges, Switzerland; 3 Departamento de Biomorfologia, Instituto de Ciências da Saúde, Universidade Federal da Bahia, Salvador, Bahia, Brazil; 4 Instituto de Investigação em Imunologia, Salvador, Bahia, Brazil; National Institutes of Health, UNITED STATES

## Abstract

**Background:**

Neutrophils are the first line of defense against invading pathogens and are rapidly recruited to the sites of *Leishmania* inoculation. During *Leishmania braziliensis* infection, depletion of inflammatory cells significantly increases the parasite load whereas co-inoculation of neutrophils plus *L*. *braziliensis* had an opposite effect. Moreover, the co-culture of infected macrophages and neutrophils also induced parasite killing leading us to ask how neutrophils alone respond to an *L*. *braziliensis* exposure. Herein we focused on understanding the interaction between neutrophils and *L*. *braziliensis*, exploring cell activation and apoptotic fate.

**Methods and Findings:**

Inoculation of serum-opsonized *L*. *braziliensis* promastigotes in mice induced neutrophil accumulation *in vivo*, peaking at 24 h. *In vitro*, exposure of thyoglycollate-elicited inflammatory or bone marrow neutrophils to *L*. *braziliensis* modulated the expression of surface molecules such as CD18 and CD62L, and induced the oxidative burst. Using mCherry-expressing *L*. *braziliensis*, we determined that such effects were mainly observed in infected and not in bystander cells. Neutrophil activation following contact with *L*. *braziliensis* was also confirmed by the release of TNF-α and neutrophil elastase. Lastly, neutrophils infected with *L*. *braziliensis* but not with *L*. *major* displayed markers of early apoptosis.

**Conclusions:**

We show that *L*. *braziliensis* induces neutrophil recruitment *in vivo* and that neutrophils exposed to the parasite *in vitro* respond through activation and release of inflammatory mediators. This outcome may impact on parasite elimination, particularly at the early stages of infection.

## Author Summary

*Leishmania* is the parasite responsible for the disease leishmaniasis, present in all continents. *Leishmania* parasites are spread through infected sand-flies and, during transmission into the vertebrate host, neutrophils are among the first cells to arrive at the infection site. Since neutrophils are key players at the frontline of defense against invading organisms, we investigated their response to *Leishmania braziliensis*. Importantly, *L*. *braziliensis* causes both Cutaneous and Mucocutaneous Leishmaniasis, two clinical manifestations characterized by their chronic development and by the presence of skin lesions with tissue destruction. Upon inoculation of mice with *L*. *braziliensis*, neutrophils rapidly arrive at the site of infection. We then observed that culture of mouse neutrophils with *L*. *braziliensis* induced the expression of adhesion molecules, production of Reactive Oxygen Species and secretion of elastase and TNF-α, two important inflammatory mediators. Also, infection with *L*. *braziliensis* induced neutrophil apoptosis, a cell death mechanism key for regulating inflammation. Our results show that neutrophils respond to presence of the *L*. *braziliensis* parasites by becoming activated and undergoing apoptosis. We suggest that this outcome modifies the local environment at the site of parasite inoculation and thus contributes with parasite killing in the infected host.

## Introduction

Neutrophils are essential components of the early inflammatory response, acting as the first line of defense against invading pathogens (rev. in [1]). Neutrophil recruitment to the infection site occurs in response to various stimuli and is followed by cell rolling and adhesion to the vasculature, processes mediated by interactions between selectins and integrins [2]. Pathogen phagocytosis subsequently elicits the production of superoxide, which is quickly dismutated into hydrogen peroxide and other secondary Reactive Oxygen Species (ROS), which are highly toxic to the invading pathogen [3]. Phagocytosis stimulates the secretion of additional antimicrobial molecules such as neutrophil elastase, into the phagosome further contributing with pathogen killing [4]. Resolution of inflammation requires efficient removal of apoptotic neutrophils by professional phagocytes such as resident macrophages [5]. Phagocytosis of apoptotic neutrophils prevents the release of potentially toxic molecules and, in parallel, regulates the inflammatory response [6].

During experimental *Leishmania* infection, neutrophils play distinct roles depending on the combination of mouse strain and parasite species. For *L*. *donovani and L*. *infantum*, neutrophils contributed to parasite killing [7,8]. For *L*. *major*, neutrophil and monocyte depletion enhanced disease in resistant mice [9–12] whereas, in susceptible mice, the absence of neutrophils inhibited Th2 cell development [12]. Neutrophil depletion led to faster lesion development in mice infected with *L*. *amazonensis* promastigotes [13] whereas amastigotes displayed resistance to the neutrophil microbicidal machinery [14]. Following phagocytosis, some *Leishmania spp* can be found within non-lytic compartments [15]. This evasion strategy suggests that *Leishmania* parasites may exploit neutrophils as to gain access to macrophages where, ultimately, infection is established [16,17]. *In vivo*, neutrophils readily arrive at the site of *L*. *major* [12,18] and *L*. *infantum-chagasi* inoculation [19] within minutes. Employing a natural transmission model, Peters *et al*. showed that neutrophils capture *L*. *major* parasites at the site of sand fly bite, but the parasites remain viable [20]. In this model, the absence of neutrophils was unfavourable to infection. More recently, Ribeiro-Gomes et al. showed that in experimental infection, the route of inoculation (intradermal, subcutaneous or intraperitoneal) also impacts on the capture of *L*. *major* parasites by neutrophils and on the establishment of infection [21].

Previously, we showed that *L*. *braziliensis*-infected macrophages co-cultured with live neutrophils display a reduced parasite load [22]. This outcome was dependent on the interaction between macrophages and neutrophils and was associated with the production of TNFα and superoxide. We suggested that clearance of neutrophils in *L*. *braziliensis*-infected mice promotes a pro-inflammatory environment, contributing with parasite clearance. Herein we investigated how exposure to *L*. *braziliensis* and internalization or not of the parasite impacts the neutrophil response.

## Methods

### Ethics statement

Female BALB/c mice, 6–8 weeks of age, were obtained from CPqGM/FIOCRUZ animal facility where they were maintained under pathogen-free conditions. All animal work was conducted according to the Guidelines for Animal Experimentation of the Colégio Brasileiro de Experimentação Animal and of the Conselho Nacional de Controle de Experimentação Animal. The local Ethics Committee on Animal Care and Utilization (CEUA) approved all procedures involving animals (L-03/2011).

### Parasites

*L*. *braziliensis* promastigotes (strain MHOM/BR/01/BA788) [23] or transgenic *L*. *braziliensis* parasites expressing mCherry [24], kindly provided by Phillip Scott (University of Pennsylvania), were grown in Schneider’s insect medium (LGC) supplemented with 10% FBS, 2 mM glutamine, 100 U/ml penicillin, and 100 mg/ml streptomycin. Parasite cultures were seeded at 10^5^ parasites/mL and were closely monitored to ascertain that parasites had reached the stationary phase (7 days). Before co-culture experiments with neutrophils, stationary-phase parasites were opsonized with 5% heat-inactivated fresh naïve serum for 30 min at 24°C. Metacyclic enriched promastigotes were obtained as described elsewhere [25]. In some experiments, we employed *L*. *major* (WHOM/IL/80/Friedlin) or dead parasites, prepared as described [26].

### Neutrophil recruitment

BALB/c mice were inoculated in the ear dermis with 10^6^ stationary phase promastigotes, in 10μL, using a 27 1/2G needle. Control mice were injected with serum-free DMEM medium. After 6, 24 and 48h post-inoculation, mice were sacrificed and the dorsal and ventral ear sheets separated with forceps. The two leaflets were transferred to RPMI supplemented with 10% FCS and antibiotics. After 16h the cells emigrating out of the ear explants were collected, counted and stained for flow cytometry [27]. For cell surface molecules, mAb 24G2 was used to block FcRs and cells were stained using anti-Ly6G-APC/Cy7 (clone 1A8) (BioLegend) and anti-CD11b-eFluor 450 (clone M1/70) (eBioscience). All cell events were acquired on an LSRII flow cytometer (BD Biosciences) and analyzed using FlowJo (Tree Star, Inc.).

### Isolation of neutrophils

Peritoneal neutrophils (herein referred as inflammatory neutrophils) were obtained by i.p. injection of 10% thioglycollate (SIGMA), as described [28]. Cells were collected 18 h later, by peritoneal washings, counted, and were left to adhere for 1h at 37°C. Non-adherent cells were recovered, washed and examined for purity by both FACS and H&E staining of cytospin preparations. Neutrophils of purity >90% were used in experiments. Bone marrow neutrophils were obtained from the tibia and femur of mice; labeled with neutrophil-specific mAbs anti-Ly6G (clone NIMP-R14-FITC or clone-1A8-PE) (BD PharMingen) and purified by MACS-positive selection, using using anti-FITC or anti-PE magnetic beads (Miltenyi Biotech). Alternatively, neutrophils were labeled with anti-Ly6G (clone 1A8, conjugated to Biotin, Miltenyi Biotech) and purified using anti-Biotin magnetic beads. Purity of neutrophils following either NIMP-R14 or 1A8 positive MACS selection was **>**95%, as assessed by FACS. Control stainings with CD11b and Ly6C were performed following magnetic separation and neutrophils (inflammatory and bone marrow) were characterized as CD11b^+^1A8^+^Ly6C^int^ and Gr1^high^ (S1 and S2 Figs, respectively).

### Neutrophil culture with *L*. *braziliensis*

Inflammatory neutrophils or bone marrow neutrophils were cultured for 2h, in RPMI medium supplemented with 10% FCS, 100 U/ml of penicillin and 100 μg/ml of streptomycin (all from Invitrogen), in the presence or absence of serum-opsonized *L*. *braziliensis* (at a 2:1 parasite:cell ratio). The infection rate of inflammatory or bone marrow neutrophils co-cultured with *L*. *braziliensis*-expressing mCherry was determined by flow cytometry. Data were acquired on a Fortessa or an LSRII flow cytometer (BD Biosciences) and analyzed using FlowJo (Tree Star Inc.).

### Expression of surface molecules and measurement of oxidative burst

Neutrophils were co-cultured *L*. *braziliensis*, as described, for 2 h. For cell surface staining, neutrophils were incubated with FcBlock (CD16/CD32) (BD Pharmingen) followed by anti-CD18-FITC (clone M18/2) or anti-CD62L-PE (clone MEL-14) (all from E-bioscience, including isotype control Rat IgG2a). For the detection of Reactive Oxygen Species, cells were co-cultured with *L*. *braziliensis* for 2 h and were later stained with Dihydroethidium (DHE) (Invitrogen), a superoxide indicator, at 3 μM for 30 minutes. As a positive control, neutrophils were incubated with phorbol 12-myristate 13-acetate (PMA) (100nM) (SIGMA) for 30 minutes. Data were acquired with a FACSAria or FACScan (BD Biosciences) and analyzed with FlowJo (Tree Star Inc.).

### Measurement of Elastase activity and of TNF-α production

Neutrophils were co-cultured with *L*. *braziliensis*, as explained above, for 4h. Elastase enzymatic activity was measured as described [10]. Briefly, cell culture supernatants were harvested and added (20 μL) in triplicate to ELISA plates. Following addition of Elastase reaction buffer (55μL) (0.1 M HEPES, 0.5 M NaCl, 10% dimethylsulfoxide, pH 7.5) and 0.2 mM Elastase substrate I (MeOSuc-AAPV-pna; Calbiochem) (150 μL), samples were incubated at 37°C for 3 days. Elastase activity was determined by reading absorbance at 410 nm, using serial dilutions of human elastase (Calbiochem), as standards. For the detection of TNF-α, neutrophils were co-cultured with *L*. *braziliensis* for 24h. Cell culture supernatants were collected and TNF-α levels were determined by ELISA, using a commercial kit (R&D Systems).

### Measurement of neutrophil apoptosis

Inflammatory neutrophils were co-cultured with *L*. *braziliensis* or *L*. *major* (at a 5:1 parasite:cell ratio) for 18 h. Neutrophils were then stained with Annexin V-FITC and PI (both from BD Biosciences). Bone marrow neutrophils were co-cultured with mCherry-*L*. *braziliensis*, as described above, for 18 h. Apoptotic neutrophils were obtained by ultraviolet irradiation exposure (245nm) for 10 minutes [10]. Cells were stained with Annexin V-FITC (Biolegend) and DAPI (SIGMA) and apoptosis was assessed by flow cytometry. Data were acquired with a FACSAria or FACScan (BD Biosciences) and analyzed with FlowJo (Tree Star. Inc.).

### Transmission electron microscopy

Inflammatory neutrophils were co-cultured with *L*. *braziliensis* or *L*. *major* (at a 5:1 parasite:cell ratio) for 18 h. Cells were fixed with 2% glutaraldehyde in 0.1 M cacodylate buffer, pH 7.4, and post-fixed in 1% OsO_4_ and 0.8% potassium ferricyanide and 5 mM calcium chloride in the same buffer. Cells were dehydrated in a graded series of acetone and embedded in Poly/Bed 812 (Polysciences, Inc.) resin. Ultrathin sections were stained with uranyl acetante and lead citrate and examined on a Zeiss109 transmission electron microscope operating at 80 KV.

### Statistical analysis

The significance of the results was calculated using non-parametrical statistical tests [Mann Whitney (two-sided t-test) or Kruskal-Wallis followed by Dunn’s post test]. Analyses were conducted using Prism (GraphPad software) and a p-value of <0.05 was considered significant.

## Results

### Neutrophil recruitment to the site of *L*. *braziliensis* inoculation

**Fig 1:**
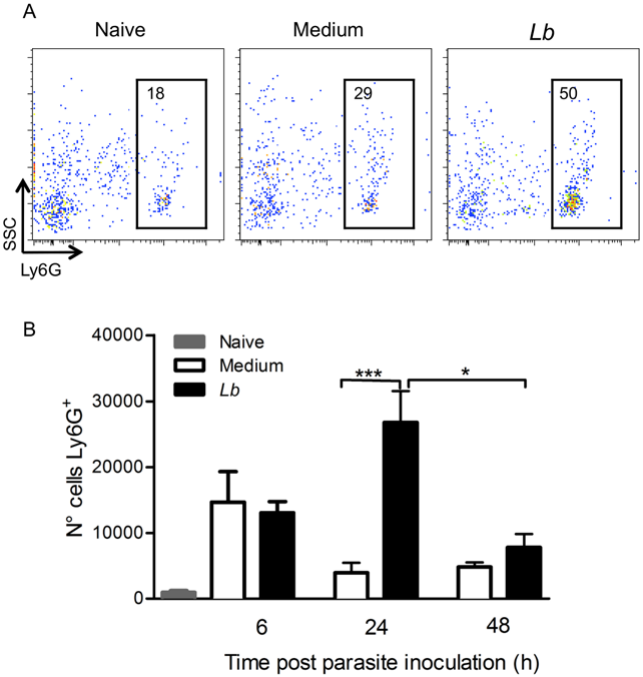
Neutrophil recruitment following *L*. *braziliensis* inoculation. Individual BALB/c mice were injected with *L*. *braziliensis* in the ear dermis. At different time points, cells were prepared and stained for Ly6G and analyzed by flow cytometry. (A) Representative dot plots and gating strategy of neutrophils. (B) Bar graph representation of the number of Ly6G^+^ cells in naïve mice (grey bars), in mice inoculated with saline (white bars) or inoculated with *L*. *braziliensis* (black bars). Data shown (mean ± SEM) are from one experiment representative of two. ***p <0.001, *p<0.05.

Previously, we reported that neutrophils are present throughout the course of lesion development in BALB/c mice inoculated with *L*. *braziliensis* [22]. Herein, we initially evaluated the kinetics of neutrophil recruitment at the early moments following *L*. *braziliensis* infection. Mice were inoculated in the ear dermis with *L*. *braziliensis* parasites and recruited cells were selected based on size and granularity; within this population, we defined Ly6G^+^ neutrophils (Fig. 1A). Six hours following parasite inoculation, we did not see differences in the number of Ly6G^+^ neutrophils comparing mice inoculated with *L*. *braziliensis* and control mice, inoculated with saline (Fig. 1B). Twenty-four hours later, the number of Ly6G^+^ cells recruited to the inoculation site significantly increased in experimental mice and 48h later, this number decreased (p<0.05, compared to the 24 h time-point). These results show that neutrophil recruitment peaks one day after *L*. *braziliensis* inoculation.

### Expression of CD62L and CD18 in *L*. *braziliensis*-exposed neutrophils

**Fig 2:**
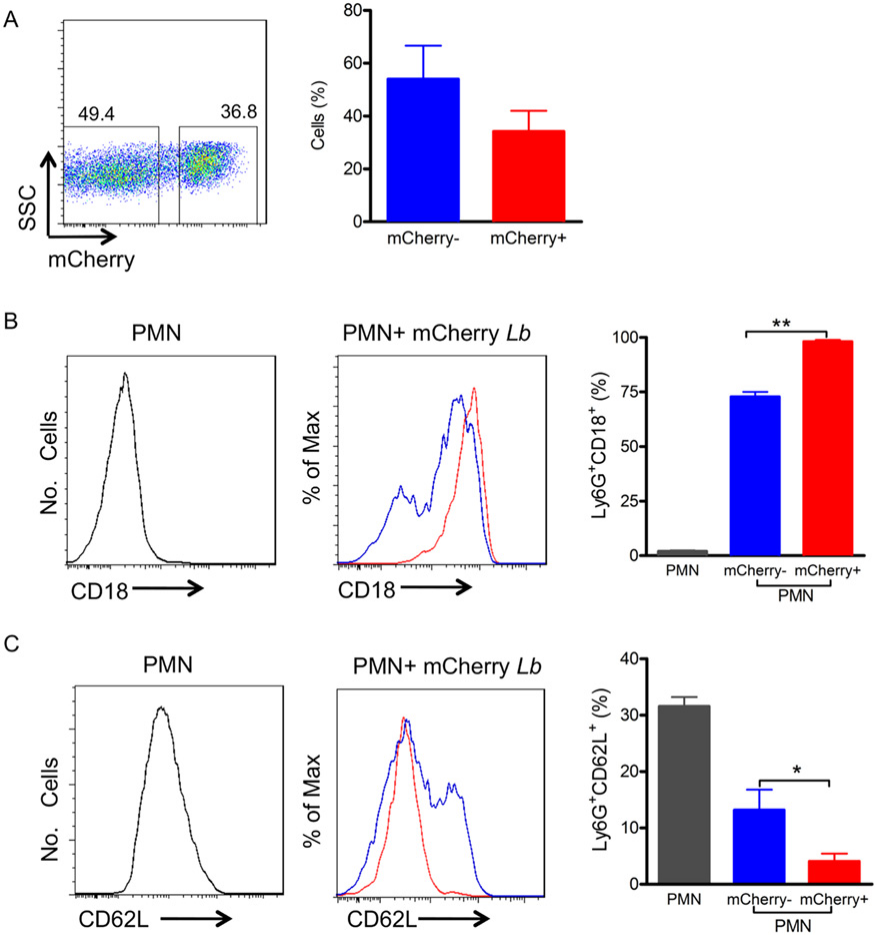
Inflammatory neutrophils infected with *L*. *braziliensis* up-regulate CD18 and down-regulate CD62L. Inflammatory neutrophils were co-cultured with mCherry *L*. *braziliensis*. (A) Representative dot plot and bar graph depicting the percentage of non-infected (mCherry^-^) and infected (mCherry^+^) neutrophils. Representative histograms depicting CD18 (B) and CD62L (C) staining in control neutrophils cultured alone (gray), bystander neutrophils (mCherry^-^) (blue, shown as% of Max) and infected neutrophils (mCherry^+^) (red, shown as% of Max). Bar graphs represent the percentages of control, bystander and infected neutrophils expressing CD18 (B) and CD62L (C). Data shown (mean ± SEM) are pooled from two independent repeats. **p<0.01; *p<0.05.

Following the observation that neutrophils rapidly accumulate at the site of *L*. *braziliensis* inoculation (Fig. 1), we investigated the expression of molecules important for cell rolling such as CD62L (L-selectin) and adherence and transmigration such as CD18 (β2 integrin). We also employed serum-opsonized *L*. *brazilensis* since *Leishmania* is delivered into the host in a blood pool, where promastigotes likely encounter serum and the complement system. Inflammatory neutrophils were co-cultured with serum-opsonized mCherry *L*. *brazilensis* and neutrophils were selected by size and granularity and, subsequently, by expression of Ly6G (Fig. 2A). In parallel, we also compared infected neutrophils (Ly6G^+^mCherry^+^) and bystander neutrophils (Ly6G^+^mCherry^-^), the latter defined as neutrophils that remained uninfected in spite of exposure to *L*. *braziliensis* (Fig. 2A). Following co-culture of inflammatory neutrophils with *L*. *braziliensis*, the percentage of infected (mCherry^+^) neutrophils was approximately 36% (Fig. 2B) whereas 49% of cells remained uninfected (mCherry^-^). The percentage of CD18^+^ cells among infected (mCherry^+^) neutrophils was higher (p<0.01) in comparison to bystanders (mCherry^-^) (Fig. 2B) whereas in control cultures (not exposed to neutrophils) the percentage of CD18^+^ cells was very low (Fig. 2B). In non-exposed neutrophils, the percentage of CD62L^+^ cells was high (Fig. 2C) and differently from CD18, the percentage of CD62L^+^ cells was lower (p<0.05) among infected (mCherry^+^) neutrophils compared to bystanders (mCherry^-^) (Fig. 2C). Incubation of inflammatory neutrophils with Zymozan did not significantly alter the percentage of CD18^+^ cells (S3 Fig).

**Fig 3:**
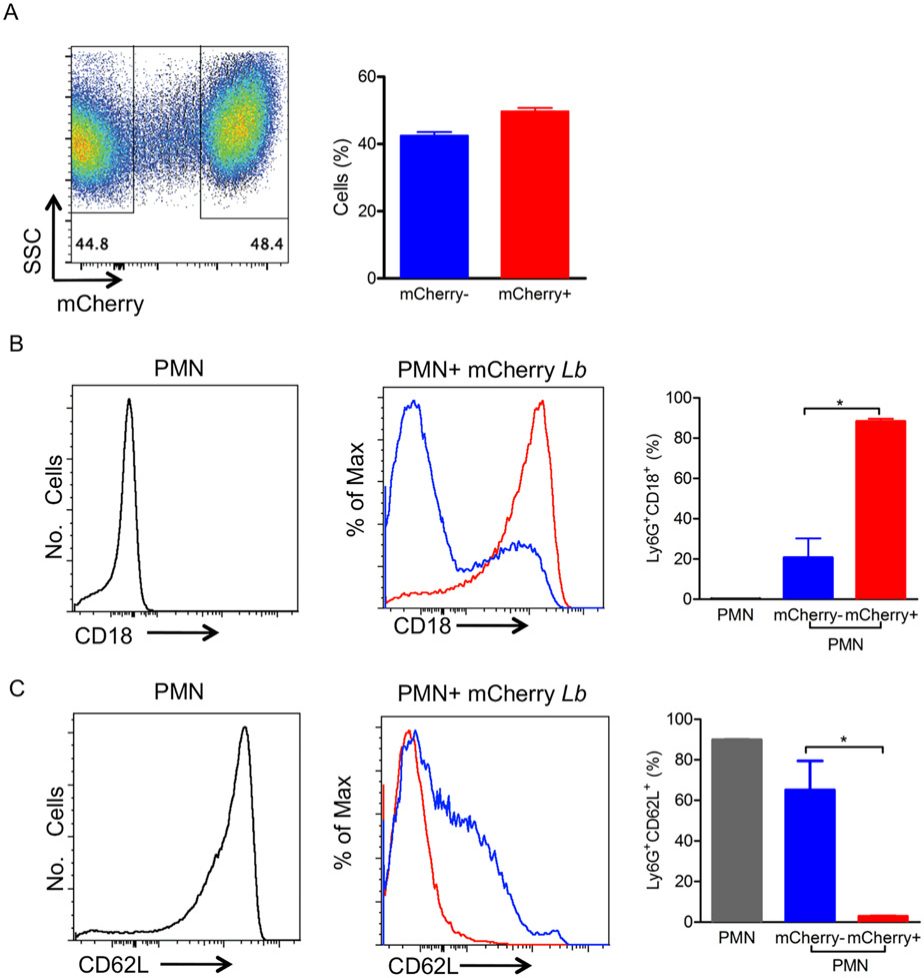
Bone-marrow neutrophils infected with *L*. *braziliensis* up-regulate CD18 and down-regulate CD62L. Bone-marrow neutrophils were co-cultured with mCherry *L*. *braziliensis*. (A) Representative dot plot and bar graph representing the percentage of non-infected (mCherry^-^) and infected (mCherry^+^) neutrophils. Representative histograms depicting CD18 (B) and CD62L (C) staining in control neutrophils cultured alone (gray), bystander neutrophils (mCherry^-^) (blue, shown as% of Max) and infected neutrophils (mCherry^+^) (red, shown as% of Max). Bar graphs represent the percentages of control, bystander and infected neutrophils expressing CD18 (B) and CD62L (C). Data shown (mean ± SEM) are pooled from two independent repeats. *p<0.05.

To expand on these findings, we performed experiments with bone marrow neutrophils, which, comparatively have an enhanced capacity to become primed [29]. Bone-marrow neutrophils were also selected by size, granularity and Ly6G expression (Fig. 3A) and following co-culture with *L*. *braziliensis*, the percentage of mCherry^+^ neutrophils was approximately 48% (Fig. 3A) whereas 43% of cells were mCherry^-^. As with inflammatory neutrophils (Fig. 2), the percentage of CD18^+^ cells was also very low in control non-exposed cultures and significantly higher (p<0.05) among infected (mCherry^+^) neutrophils compared to bystanders (mCherry^-^) (Fig. 3B). Also replicating our findings with inflammatory neutrophils (Fig. 2), the percentage of CD62L^+^ cells was highest in non-exposed neutrophils (Fig. 3C), and significantly higher (p<0.05) in bystanders (mCherry^-^) compared to infected (mCherry^+^) neutrophils. These data indicate that neutrophils infected with *L*. *braziliensis* upregulate CD18 and downregulate CD62L, regardless of their activation state.

### Production of superoxide in neutrophils exposed to *L*. *braziliensis*

**Fig 4:**
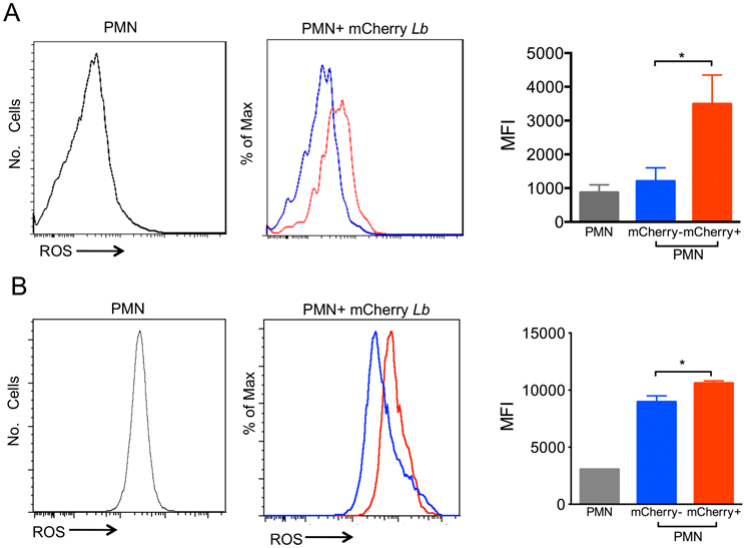
Exposure to *L*. *braziliensis* induces ROS production. Neutrophils were co-cultured with mCherry *L*. *braziliensis*, cells were stained with DHE and ROS production was analyzed by FACS. (A) Histograms represent inflammatory neutrophils cultured alone (gray), bystander neutrophils (mCherry^-^) (blue) and infected neutrophils (mCherry^+^) (red). (B) Histograms represent bone marrow neutrophils cultured alone (gray), bystander neutrophils (mCherry^-^) (blue) and infected neutrophils (mCherry^+^). Bar graphs represent the MFI of neutrophils cultured alone (PMN), bystanders (mCherry^-^) (blue) or *L*. *braziliensis*-infected (mCherry^+^) (red). Data (mean ± SEM) are from one experiment representative of two. *p<0.05.

**Fig 5:**
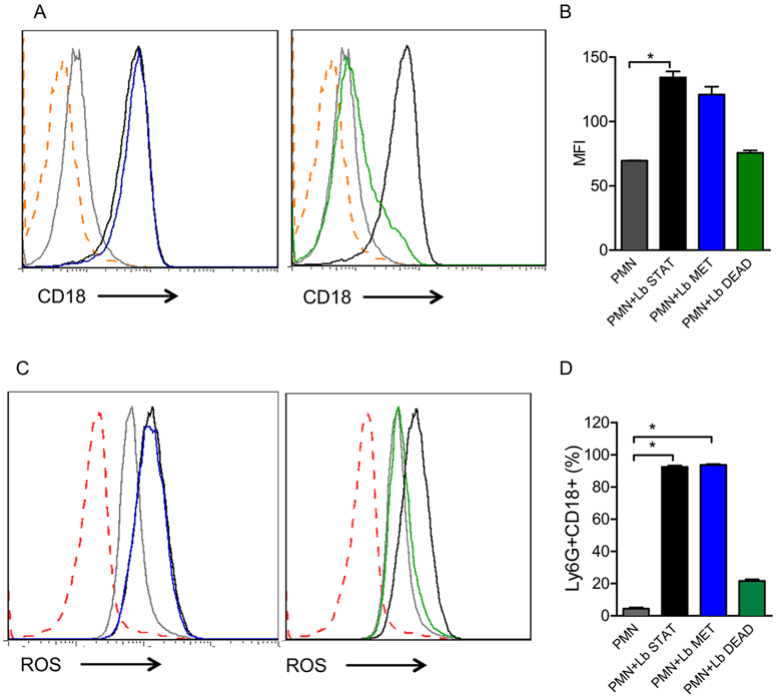
Exposure to dead *L*. *braziliensis* does not modulate CD18 expression or ROS production. Inflammatory neutrophils were co-cultured with *L*. *braziliensis* and stained with anti-CD62L. (A,C) Representative histograms depicting ROS production or CD18 staining in neutrophils cultured alone (gray), neutrophils exposed to stationary *L*. *braziliensis* (black), metacyclic *L*. *braziliensis* (blue) or dead *L*. *braziliensis* (green). Dotted orange line depicts unstained neutrophils. (B, D). Bar graphs represent the percentage of neutrophils positive for CD18. Data shown (mean ± SEM) are from one experiment representative of two. *p<0.05.

Neutrophils produce Reactive Oxygen Species (ROS), which form a central component of the defense mechanism against foreign pathogens during infection. Inflammatory neutrophils co-cultured with *L*. *braziliensis* displayed a significant increase in superoxide production (Fig. 4A), which was attributed mostly to neutrophils harboring *L*. *braziliensis*-mCherry. With bone marrow neutrophils, superoxide production was also significantly higher in cells harboring mCherry, however, ROS was also observed in bystanders (mCherry-) (Fig. 4B). Additionally, superoxide production was similar upon co-culture of inflammatory neutrophils with either stationary phase or metacyclic *L*. *braziliensis* (Fig. 5A and B). Co-culture with dead parasites also did not change superoxide production in relation to neutrophils cultured alone (Fig. 5A and B). Similar results were obtained regarding the percentage of CD18^+^ cells (Fig. 5C and D).

### Production of TNF-α and elastase by neutrophils exposed to *L*. *braziliensis*

**Fig 6:**
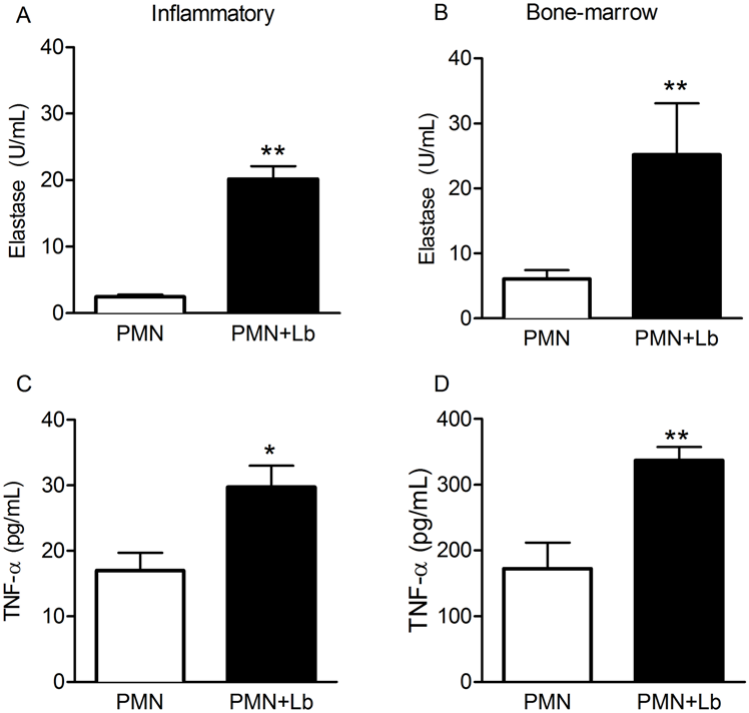
Production of Elastase and TNF-a by *L*. *braziliensis-*exposed neutrophils. Inflammatory or bone marrow neutrophils were co-cultured with *L*. *braziliensis* for 24h. Culture supernatants were assayed for the presence of TNF-α (A,B) and for the presence of free elastase activity (C,D). Data shown (mean ± SEM) are pooled from two independent repeats. **p <0.01, *p<0.05.

Neutrophils display granules enriched with antimicrobial molecules, including serine proteases such as elastase [30]. Additionally, cytokines secreted by neutrophils, such as TNF-α, influence macrophage and dendritic cell function, with important effects on the adaptive immune response [28]. Herein, co-culture with *L*. *braziliensis*, triggered the release of elastase by both inflammatory (Fig. 6A) and bone marrow neutrophils (Fig. 6B). In the same manner, the presence of TNF-α was significantly higher in cultures of inflammatory (Fig. 6C) and bone marrow neutrophils (Fig. 6D) co-cultured with *L*. *braziliensis*. We did not detect IL-10 nor IL-12p40 in the culture supernatants.

### Neutrophil apoptosis following exposure to *L*. *braziliensis*

**Fig 7:**
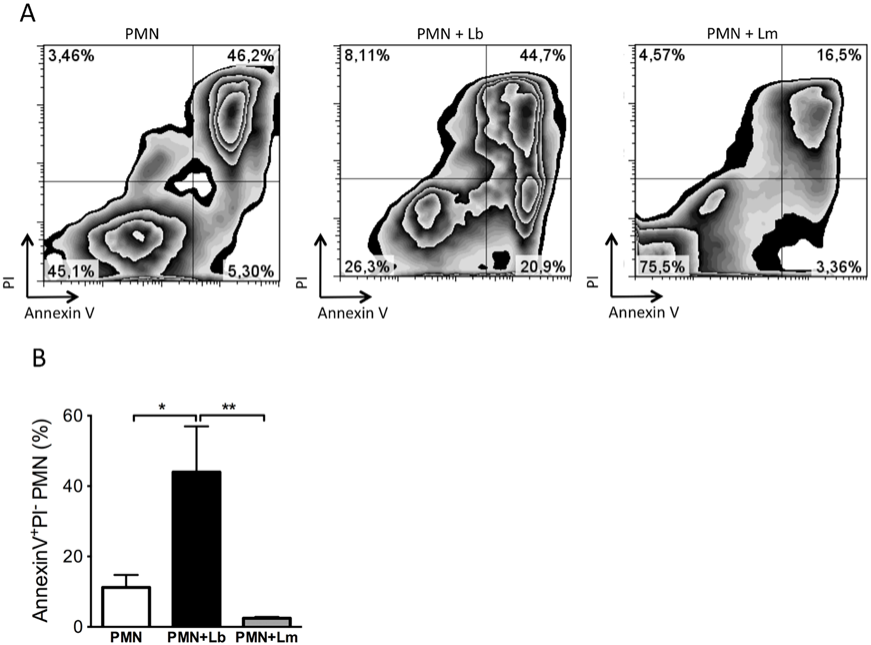
Exposure to *L*. *braziliensis* but not to *L*. *major* triggers neutrophils apoptosis. Inflammatory neutrophils were co-cultured with *L*. *braziliensis*. After 18h, neutrophils were labeled with Annexin V and PI and analyzed by FACS. (A) Representative zebra plots of Annexin V^+^/PI^+^ neutrophils (PMN) and of neutrophils co-cultured with *L*. *braziliensis* (PMN+Lb) or with *L*. *major* (PMN+Lm). (B) Percentage of Annexin V^+^/PI^-^ neutrophils alone (PMN) or neutrophils cultured with parasites (PMN+Lb and PMN+Lm). Data shown (mean ± SEM) are pooled from two independent repeats. *p<0.05; **p <0.01.

**Fig 8:**
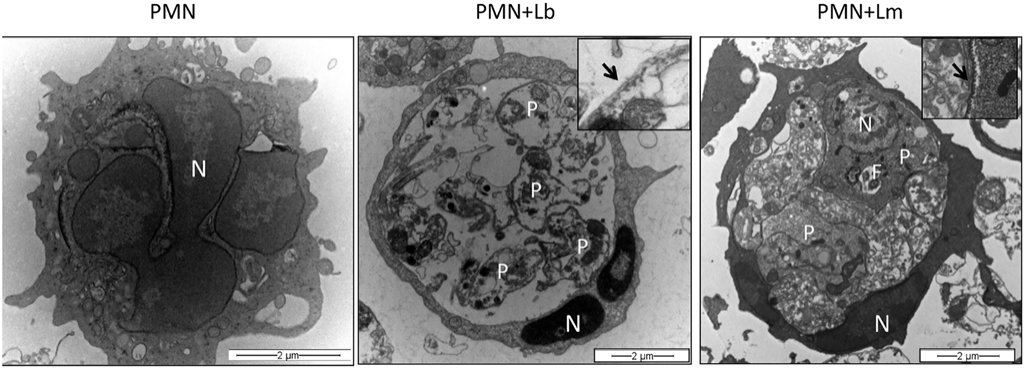
Ultrastructural analysis of neutrophils infected with *L*. *braziliensis* or *L*. *major*. Inflammatory neutrophils were co-cultured with *L*. *braziliensis* (at a 5:1 parasite:cell ratio), for 18 h. Cells were fixed with 2% glutaraldehyde in 0.1 M cacodylate buffer, pH 7.4, and post-fixed in 1% OsO_4_ and 0.8% potassium ferricyanide and 5 mM calcium chloride in the same buffer. Cells were dehydrated in a graded series of acetone and embedded in Poly/Bed 812 (Polysciences, Inc.) resin. Ultrathin sections were stained with uranyl acetante and lead citrate and examined on a Zeiss109 transmission electron microscope operating at 80 KV. (A) Uninfected neutrophils exhibiting multilobular nucleus (N). (B) Neutrophil infected with *L*. *braziliensis* showing condensed nucleus (N) and unpreserved intracellular parasite structures (P). Insert shows *Leishmania* microtubule (arrow). (C) Neutrophil infected with *L*. *major* showing preserved parasite structures (P) and nucleus (N). Insert shows *Leishmania* microtubule (arrow).

At infection sites, cells dying by apoptosis express phosphatidylserine (PS) and PS exposure can be detected by Annexin V staining and quantified by flow cytometry. Inflammatory neutrophils were co-cultured for 18 h with *L*. *braziliensis* parasites and we investigated whether this interaction resulted in apoptosis. In these co-cultures, there was a significant (p<0.05) increase in the percentage of early apoptotic (Annexin V^+^/PI^-^) neutrophils (Fig. 7A and B), compared to neutrophils cultured alone, whereas the percentage of late apoptotic/necrotic (Annexin V^+^/PI^+^) neutrophils was similar (Fig. 7A and B). On the other hand, upon co-culture with *L*. *major*, we detected a lower percentage of both early (Annexin V^+^/PI^-^) and late apoptotic/necrotic (Annexin V^+^/PI^+^) neutrophils, compared to neutrophils cultured with *L*. *braziliensis* (Fig. 7A and B). Analysis of neutrophils by transmission electron microscopy confirmed apoptosis of *L*. *braziliensis*-infected neutrophils as seen by the presence of pyknosis, chromatin condensation as well as remnants of internalized degenerated parasites (Fig. 8). Moreover, internalized *L*. *braziliensis* parasites presented chromatin condensation, cytoplasmic disorganization and vacuolization (Fig. 8). In co-cultures performed with *L*. *major*, however, the neutrophils remained with a well preserved cytoplasm and viable parasite were observed inside the parasitophorous vacuole (Fig. 8), reinforcing the finding that *L*. *major* delays neutrophil apoptosis [31], differently from *L*. *braziliensis*.

**Fig 9:**
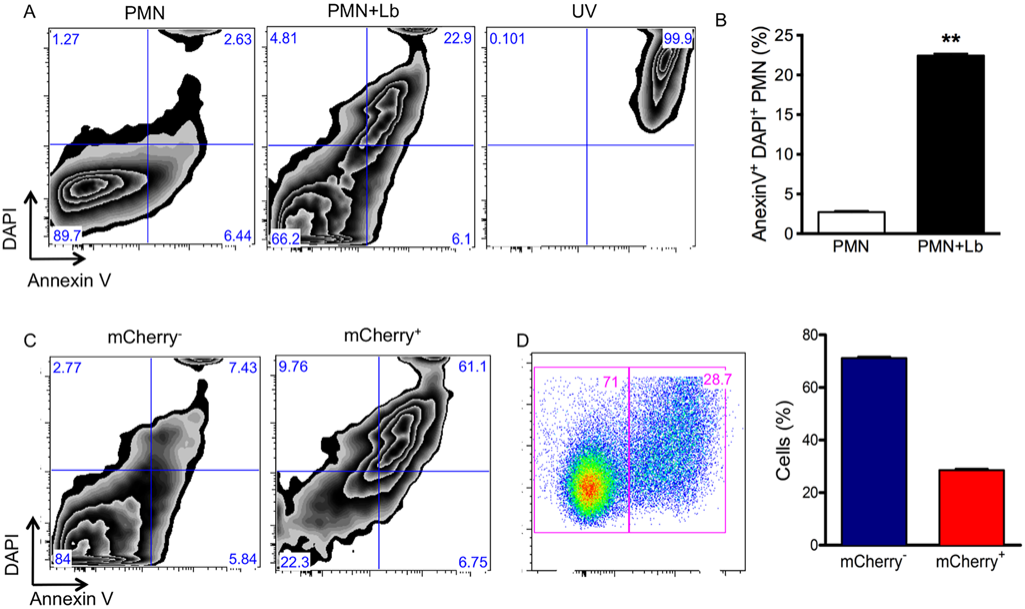
Apoptosis in neutrophils infected with *L*. *braziliensis*. Bone-marrow neutrophils were co-cultured with *L*. *braziliensis*. After 18h, neutrophils were labeled with Annexin V and DAPI and analyzed by FACS. (A) Representative zebra plots of neutrophils (PMN), neutrophils cultured with *L*. *braziliensis* (PMN+Lb) or neutrophils exposed to UV (UV). (B) Percentage of DAPI^+^/Annexin V^+^ neutrophils (PMN) or neutrophils cultivated with *L*. *braziliensis* (PMN+Lb). (C) Representative zebra plots of bystander neutrophils (mCherry-) and of infected neutrophils (mCherry^+^) expressing DAPI and Annexin V. (D) Representative dot plots of neutrophils infected with mCherry *L*. *braziliensis* after 18h, bar graph represents the percentage of non-infected (mCherry^-^) and infected (mCherry^+^) neutrophils. Data (mean ± SEM) are from one experiment representative of three experiments. **p <0.01.

Following the observation that *L*. *braziliensis* induced apoptosis in inflammatory neutrophils, we then examined whether this would also occur with bone marrow neutrophils. Indeed, upon co-culture with *L*. *braziliensis*, a significant increase in the percentage of late apoptotic/necrotic (Annexin V^+^/DAPI^+^) neutrophils (Fig. 9A and B) was observed. As a control of late apoptosis/necrosis, neutrophil exposure to UV increased the percentage of cells positive for Annexin V^+^/DAPI^+^. Importantly, late apoptosis/necrosis (AnnexinV^+^/DAPI^+^) was mostly detected in infected neutrophils (mCherry^+^) when compared with bystanders (mCherry^-^) (Fig. 9C). At this time point, the percentage of mCherry^+^ neutrophils was ~28% (Fig. 9D).

## Discussion

Numerous studies have demonstrated that neutrophils play a crucial role in immunity against bacterial, fungal [1] and intracellular pathogens [32]. Earlier on, we demonstrated that *L*. *braziliensis* inoculation into the ear dermis of BALB/c mice leads to the development of a cutaneous ulcer, which heals spontaneously after ten weeks of infection [23]. Additionally, co-inoculation of *L*. *braziliensis* and neutrophils decreased lesion size whereas depletion of neutrophils and monocytes had an opposing effect, significantly increasing parasite load and lesion size [22]. Given that neutrophils are among the first cells to encounter the parasite at the site of the sand fly bite [20] and, thus, will readily encounter *Leishmania* parasites, the purpose of the current study was to investigate how neutrophils respond to *L*. *braziliensis* exposure, evaluating neutrophil activation and downstream events such as apoptosis.

Sand flies probe the human host to obtain blood and, in this process, lacerate capillaries forming a blood pool into which *Leishmania* promastigotes are inoculated. Following this event, there is rapid accumulation of neutrophils [20] and it has been shown that the co-inoculated salivary molecules can modulate neutrophil function [33,34]. Herein, we confirmed neutrophil infiltration to the site of *L*. *braziliensis* inoculation by syringe and showed maximal accumulation at 24h. Of interest, syringe inoculation of *L*. *amazonensis*, also induced maximal neutrophil accumulation at 24 h [13], indicating a common kinetic for neutrophil recruitment for these two New World *Leishmania* species.

Following our observation that neutrophils are recruited in response to *L*. *braziliensis* inoculation, we then performed a series of *in vitro* experiments to investigate how neutrophils respond to this type of stimulation and, in addition, we compared the responses of inflammatory and bone marrow neutrophils. Initially, we evaluated the expression of adhesion molecules. β_2_ integrins are leukocyte-specific integrins required for neutrophil adhesion and transmigration across the activated endothelium [35] and CD18 is the common β_2_ integrin present in LFA1 (CD11aCD18), Mac-1/ CR3 (CD11bCD18) and p150/94/CR4 (CD11cCD18). In the presence of *L*. *braziliensis* we detected an increase in the percentage of neutrophils (inflammatory and bone marrow) expressing CD18 and this increase was associated with infected neutrophils (mCherry^+^), indicating that *L*. *braziliensis* were readily internalized. Indeed, Mac-1/CR3 (CD11bCD18) plays a major role in the phagocytosis of complement-opsonized *L*. *major* promastigotes by both macrophages [36–38] and human neutrophils [39].

L-selectin (CD62L) participates in neutrophil tethering and rolling [40] but it is cleaved from the leukocyte surface following cellular activation and exposure to inflammatory stimuli [41,42]. Upon co-culture with *L*. *braziliensis*, we detected a lower percentage of inflammatory CD62L^+^/mCherry^+^ neutrophils, compared to bystanders (mCherry^-^). Similar results were obtained with bone marrow neutrophils, indicating that *L*. *braziliensis* phagocytosis induced more CD62L shedding, marking neutrophil activation [43]. With regards to bystanders, the percentage of inflammatory CD62L^+^/mCherry^-^ neutrophils was lower compared to bone marrow (CD62L^+^/mCherry^-^) neutrophils, possibly reflecting their already primed nature and their extravasation to the peritoneum following thyoglycollate stimulation. Such difference may also be related to the priming potential of bone marrow neutrophils vs. inflammatory, as shown by fMLP stimulation and induction of ROS [29]. Also, we cannot presently attribute CD62L shedding to the infection rate since mCherry staining was similar for both bone marrow (~48%) and inflammatory (~36%) neutrophils. Although we do not know which molecules may be activating bystander neutrophils, it has been shown that *L*. *amazonensis* LPG activates human neutrophils in levels similar to those observed with promastigotes [44].

In the presence of *L*. *braziliensis*, both inflammatory and bone marrow neutrophils displayed a significant increase in the production of superoxide, a hallmark of neutrophil activation, and ROS detection was significantly higher in infected (mCherry+) neutrophils. Similar results were obtained in experiments with other *Leishmania* spp. [14,44–46]. Neutrophils exposed to ROS also up-regulate the production of TNF-α and MIP-2 [47,48] and TNF-α primes murine neutrophils to become activated, an effect that is concomitant with the mobilization of CR3-containing granules to the plasma membrane [49]. Since TNF-α and CD18 expression were increased upon neutrophil-co-culture with *L*. *braziliensis*, we can suggest that ROS produced by infected cells contributed with TNF-α secretion and CD18 (a Mac1/CR3 component) expression. Furthermore, elastase production was also elevated in neutrophils cultured with *L*. *braziliensis* and, importantly, elastase was associated with the killing of intracellular *Leishmania* in macrophages cultured with neutrophils [10], a process dependent on TLR4 signaling [50]. IL-10 production, on the other hand, was not modulated in our experiments, as seen in previous studies [14,51].

Cell death and the subsequent clearance of apoptotic neutrophils is crucial for maintaining homeostasis and, at the same time, necessary for resolution of inflammation. At inflammatory sites, neutrophils can undergo spontaneous apoptosis [52] or apoptosis due to the recognition of cell-death mediators such as TNF-α and FasL [53]. Co-culture with *L*. *braziliensis* induced neutrophil apoptosis, findings that were confirmed by transmission electron microscopy analysis. Indeed, infected neutrophils displayed condensed chromatin and degraded intracellular parasites. Similar results were obtained with bone marrow neutrophils: Annexin^+^/DAPI^+^ staining was significantly higher in infected cells (mCherry^+^) compared to bystanders (mCherry^-^) and the percentage of infected neutrophils (mCherry^+^) was lower compared to bystanders (mCherry^-^). We can suggest that phagocytosis of *L*. *braziliensis* results in apoptosis and, in parallel, parasite destruction, hence the lower percentage of infected cells. In addition, ROS [54] and TNF-α [55] also trigger neutrophil apoptosis, two mediators that were produced upon culture with *L*. *braziliensis*.

Neutrophil apoptosis was also observed upon culture of neutrophils with *L*. *amazonensis* [14] but *L*. *major*, on the other hand, delays neutrophil apoptosis [31], enhancing cell lifespan [56]. Parasites survive within infected neutrophils [39,57] and viable parasites have been recovered by cell sorting [20]. Indeed, in our hands, the frequency of late apoptotic (Annexin^+^/PI^+^) staining was low in neutrophils cultured with *L*. *major* in contrast to neutrophils cultured alone and to neutrophils cultured with *L*. *braziliensis*, both of which were positive for Annexin/PI (Fig. 6). Electron microscopy confirmed the presence remnants of *L*. *braziliensis* parasites while in contrast, intact parasites were found within *L*. *major* infected neutrophils (Fig. 7). Clearance of apoptotic neutrophils by macrophages promotes parasite replication in vitro [58], indicating that *L*. *major* may exploit neutrophil apoptosis as means to ascertain infection. Moreover, the phagocytosis of apoptotic neutrophils inhibits the response to *L*. *major* [59]. Therefore, for *L*. *major*, current literature indicates that neutrophils are rapidly and massively recruited to the site of *Leishmania* inoculation, where they phagocytose the parasites. Depending on the source of neutrophils, species and strains of *Leishmania*, internalized parasites can survive and neutrophils would thus provide a transient safe shelter prior to parasite entry into macrophages, the definitive host cell (rev. in [17,60]). In experiments with *L*. *braziliensis*, however, co-culture of infected macrophages with UV-treated neutrophils did not modulate the parasite load [22], also suggesting that differences within *Leishmania* species may induce distinct outcomes regarding neutrophil apoptosis and downstream effects.

We showed that neutrophils are recruited to the site of *L*. *brazilensis* inoculation and upon contact with promastigotes, *in vitro*, neutrophils become activated producing superoxide, TNF-α and elastase. Later, we observed neutrophil apoptosis, particularly of infected cells. However, once amastigotes become predominant, a different scenario may ensue since this stage is more resistant to these same effector mechanisms, as recently described for *L*. *amazonensis* [14], impacting on disease development. Indeed, BALB/c mice infected with *L*. *braziliensis* develop cutaneous ulcers, despite the presence of neutrophils [23]. However, in this experimental model lesions heal spontaneously and parasites are eliminated from the infection site. Neutrophils could also play a role at the chronic stages of infection, through cooperation with *L*. *braziliensis*-infected macrophages, as previously shown in vitro [22]. Neutrophils have been shown to cross-talk with dendritic cells [27,61,62] and such cross talk may also be related to the development of the adaptive immune response to *L*. *braziliensis*. However this remains to be investigated. Thus, the strong impact of *L*. *braziliensis* on neutrophils phenotype and function reported here *in vitro* are likely to occur at the onset of infection with the parasite, suggesting that these cells are playing a crucial role following infection.

## Supporting Information

S1 Fig Characterization of inflammatory neutrophils by flow cytometry. Inflammatory neutrophils were purified using MACS and the 1A8 (Ly6G) mAb. In this sample cells were first gated for size and granularity (SSC x FSC). The gated cells were further analyzed for expression of CD11b/1A8 or 1A8/Ly6C.
(TIF)

S2 Fig Characterization of bone marrow neutrophils by flow cytometry. Bone marrow neutrophils were purified using MACS and the 1A8 (Ly6G) mAb. In this sample cells were first gated for size and granularity (SSC x FSC). The gated cells were further analyzed for expression of CD11b/1A8 or Gr-1/Ly6C.
(TIF)

S3 Fig Modulation in CD18 expression is associated with *L*. *braziliensis* exposure. Inflammatory neutrophils were co-cultured with *L*. *braziliensis* or with Zymozan (SIGMA (100ug/ml). (A) Representative dot plots showing the gating strategy used to identify neutrophils (Ly6G) following exposure to *L*. *braziliensis*. Cells were stained with anti-CD18 (B) and were analyzed by FACS. Orange histograms: isotype control. Gray histogram: neutrophils cultured in medium only. Red histograms: neutrophils cultured with Zymozan. Black histograms, neutrophils exposed to *L*. *braziliensis*. Bar graphs represent the percentage of neutrophils positive for CD18. Data shown (mean ± SEM) are pooled from two independent repeats. *p<0.05 (One Way ANOVA).
(TIF)

## References

[1] Nathan C (2006) Neutrophils and immunity: challenges and opportunities. Nat Rev Immunol 6: 173–182. 16498448 10.1038/nri1785

[2] Zarbock A, Ley K (2008) Mechanisms and consequences of neutrophil interaction with the endothelium. Am J Pathol 172: 1–7. 18079440 10.2353/ajpath.2008.070502 PMC2189633

[3] Babior BM (1999) NADPH oxidase: an update. Blood 93: 1464–1476. 10029572

[4] Borregaard N, Sorensen OE, Theilgaard-Monch K (2007) Neutrophil granules: a library of innate immunity proteins. Trends Immunol 28: 340–345. 17627888 10.1016/j.it.2007.06.002

[5] Savill J, Haslett C (1995) Granulocyte clearance by apoptosis in the resolution of inflammation. Semin Cell Biol 6: 385–393. 8748146 10.1016/s1043-4682(05)80009-1

[6] Fadok VA, Bratton DL, Konowal A, Freed PW, Westcott JY, et al (1998) Macrophages that have ingested apoptotic cells in vitro inhibit proinflammatory cytokine production through autocrine/paracrine mechanisms involving TGF-beta, PGE2, and PAF. J Clin Invest 101: 890–898. 9466984 10.1172/JCI1112 PMC508637

[7] McFarlane E, Perez C, Charmoy M, Allenbach C, Carter KC, et al (2008) Neutrophils contribute to development of a protective immune response during onset of infection with Leishmania donovani. Infect Immun 76: 532–541. 18056477 10.1128/IAI.01388-07 PMC2223441

[8] Rousseau D, Demartino S, Ferrua B, Michiels JF, Anjuere F, et al (2001) In vivo involvement of polymorphonuclear neutrophils in Leishmania infantum infection. BMC Microbiol 1: 17 11591218 10.1186/1471-2180-1-17 PMC57739

[9] Lima GM, Vallochi AL, Silva UR, Bevilacqua EM, Kiffer MM, et al (1998) The role of polymorphonuclear leukocytes in the resistance to cutaneous Leishmaniasis. Immunol Lett 64: 145–151. 9870666 10.1016/s0165-2478(98)00099-6

[10] Ribeiro-Gomes FL, Otero AC, Gomes NA, Moniz-De-Souza MC, Cysne-Finkelstein L, et al (2004) Macrophage interactions with neutrophils regulate Leishmania major infection. J Immunol 172: 4454–4462. 15034061 10.4049/jimmunol.172.7.4454

[11] Chen L, Zhang ZH, Watanabe T, Yamashita T, Kobayakawa T, et al (2005) The involvement of neutrophils in the resistance to Leishmania major infection in susceptible but not in resistant mice. Parasitol Int 54: 109–118. 15866472 10.1016/j.parint.2005.02.001

[12] Tacchini-Cottier F, Zweifel C, Belkaid Y, Mukankundiye C, Vasei M, et al (2000) An immunomodulatory function for neutrophils during the induction of a CD4+ Th2 response in BALB/c mice infected with Leishmania major. J Immunol 165: 2628–2636. 10946291 10.4049/jimmunol.165.5.2628

[13] Sousa LM, Carneiro MB, Resende ME, Martins LS, Dos Santos LM, et al (2014) Neutrophils have a protective role during early stages of Leishmania amazonensis infection in BALB/c mice. Parasite Immunol 36: 13–31. 10.1111/pim.12078 24102495 PMC4307027

[14] Carlsen ED, Hay C, Henard CA, Popov V, Garg NJ, et al (2013) Leishmania amazonensis amastigotes trigger neutrophil activation but resist neutrophil microbicidal mechanisms. Infect Immun 81: 3966–3974. 10.1128/IAI.00770-13 23918780 PMC3811833

[15] Gueirard P, Laplante A, Rondeau C, Milon G, Desjardins M (2008) Trafficking of Leishmania donovani promastigotes in non-lytic compartments in neutrophils enables the subsequent transfer of parasites to macrophages. Cell Microbiol 10: 100–111. 17651446 10.1111/j.1462-5822.2007.01018.x

[16] Laskay T, van Zandbergen G, Solbach W (2003) Neutrophil granulocytes—Trojan horses for Leishmania major and other intracellular microbes? Trends Microbiol 11: 210–214. 12781523 10.1016/s0966-842x(03)00075-1

[17] Laskay T, van Zandbergen G, Solbach W (2008) Neutrophil granulocytes as host cells and transport vehicles for intracellular pathogens: Apoptosis as infection-promoting factor. Immunobiology 213: 183–191. 10.1016/j.imbio.2007.11.010 18406366

[18] Muller K, van Zandbergen G, Hansen B, Laufs H, Jahnke N, et al (2001) Chemokines, natural killer cells and granulocytes in the early course of Leishmania major infection in mice. Med Microbiol Immunol 190: 73–76. 11770115 10.1007/s004300100084

[19] Thalhofer CJ, Chen Y, Sudan B, Love-Homan L, Wilson ME (2011) Leukocytes infiltrate the skin and draining lymph nodes in response to the protozoan Leishmania infantum chagasi. Infect Immun 79: 108–117. 10.1128/IAI.00338-10 20937764 PMC3019875

[20] Peters NC, Egen JG, Secundino N, Debrabant A, Kimblin N, et al (2008) In vivo imaging reveals an essential role for neutrophils in leishmaniasis transmitted by sand flies. Science 321: 970–974. 10.1126/science.1159194 18703742 PMC2606057

[21] Ribeiro-Gomes FL, Roma EH, Carneiro MB, Doria NA, Sacks DL, et al (2014) Site-Dependent Recruitment of Inflammatory Cells Determines the Effective Dose of Leishmania major. Infect Immun 82: 2713–2727. 10.1128/IAI.01600-13 24733090 PMC4097609

[22] Novais FO, Santiago RC, Bafica A, Khouri R, Afonso L, et al (2009) Neutrophils and macrophages cooperate in host resistance against Leishmania braziliensis infection. J Immunol 183: 8088–8098. 10.4049/jimmunol.0803720 19923470

[23] de Moura TR, Novais FO, Oliveira F, Clarencio J, Noronha A, et al (2005) Toward a novel experimental model of infection to study American cutaneous leishmaniasis caused by Leishmania braziliensis. Infect Immun 73: 5827–5834. 16113301 10.1128/IAI.73.9.5827-5834.2005 PMC1231065

[24] Novais FO, Carvalho LP, Graff JW, Beiting DP, Ruthel G, et al (2013) Cytotoxic T cells mediate pathology and metastasis in cutaneous leishmaniasis. PLoS Pathog 9: e1003504 10.1371/journal.ppat.1003504 23874205 PMC3715507

[25] Spath GF, Beverley SM (2001) A lipophosphoglycan-independent method for isolation of infective Leishmania metacyclic promastigotes by density gradient centrifugation. Exp Parasitol 99: 97–103. 11748963 10.1006/expr.2001.4656

[26] Okwor I, Liu D, Beverley SM, Uzonna JE (2009) Inoculation of killed Leishmania major into immune mice rapidly disrupts immunity to a secondary challenge via IL-10-mediated process. Proc Natl Acad Sci U S A 106: 13951–13956. 10.1073/pnas.0905184106 19666482 PMC2729001

[27] Charmoy M, Brunner-Agten S, Aebischer D, Auderset F, Launois P, et al (2010) Neutrophil-derived CCL3 is essential for the rapid recruitment of dendritic cells to the site of Leishmania major inoculation in resistant mice. PLoS Pathog 6: e1000755 10.1371/journal.ppat.1000755 20140197 PMC2816696

[28] Bennouna S, Denkers EY (2005) Microbial antigen triggers rapid mobilization of TNF-alpha to the surface of mouse neutrophils transforming them into inducers of high-level dendritic cell TNF-alpha production. J Immunol 174: 4845–4851. 15814711 10.4049/jimmunol.174.8.4845

[29] Itou T, Collins LV, Thoren FB, Dahlgren C, Karlsson A (2006) Changes in activation states of murine polymorphonuclear leukocytes (PMN) during inflammation: a comparison of bone marrow and peritoneal exudate PMN. Clin Vaccine Immunol 13: 575–583. 16682479 10.1128/CVI.13.5.575-583.2006 PMC1459655

[30] Faurschou M, Borregaard N (2003) Neutrophil granules and secretory vesicles in inflammation. Microbes Infect 5: 1317–1327. 14613775 10.1016/j.micinf.2003.09.008

[31] Aga E, Katschinski DM, van Zandbergen G, Laufs H, Hansen B, et al (2002) Inhibition of the spontaneous apoptosis of neutrophil granulocytes by the intracellular parasite Leishmania major. J Immunol 169: 898–905. 12097394 10.4049/jimmunol.169.2.898

[32] Appelberg R (2007) Neutrophils and intracellular pathogens: beyond phagocytosis and killing. Trends Microbiol 15: 87–92. 17157505 10.1016/j.tim.2006.11.009

[33] Prates DB, Araujo-Santos T, Luz NF, Andrade BB, Franca-Costa J, et al (2011) Lutzomyia longipalpis saliva drives apoptosis and enhances parasite burden in neutrophils. J Leukoc Biol 90: 575–582. 10.1189/jlb.0211105 21685247

[34] Chagas AC, Oliveira F, Debrabant A, Valenzuela JG, Ribeiro JM, et al (2014) Lundep, a sand fly salivary endonuclease increases Leishmania parasite survival in neutrophils and inhibits XIIa contact activation in human plasma. PLoS Pathog 10: e1003923 10.1371/journal.ppat.1003923 24516388 PMC3916414

[35] Coxon A, Rieu P, Barkalow FJ, Askari S, Sharpe AH, et al (1996) A novel role for the beta 2 integrin CD11b/CD18 in neutrophil apoptosis: a homeostatic mechanism in inflammation. Immunity 5: 653–666. 8986723 10.1016/s1074-7613(00)80278-2

[36] Mosser DM, Edelson PJ (1985) The mouse macrophage receptor for C3bi (CR3) is a major mechanism in the phagocytosis of Leishmania promastigotes. J Immunol 135: 2785–2789. 3161950

[37] Wozencraft AO, Blackwell JM (1987) Increased infectivity of stationary-phase promastigotes of Leishmania donovani: correlation with enhanced C3 binding capacity and CR3-mediated attachment to host macrophages. Immunology 60: 559–563. 2953670 PMC1453286

[38] Rosenthal LA, Sutterwala FS, Kehrli ME, Mosser DM (1996) Leishmania major-human macrophage interactions: cooperation between Mac-1 (CD11b/CD18) and complement receptor type 1 (CD35) in promastigote adhesion. Infect Immun 64: 2206–2215. 8675328 10.1128/iai.64.6.2206-2215.1996 PMC174057

[39] Laufs H, Muller K, Fleischer J, Reiling N, Jahnke N, et al (2002) Intracellular survival of Leishmania major in neutrophil granulocytes after uptake in the absence of heat-labile serum factors. Infect Immun 70: 826–835. 11796617 10.1128/iai.70.2.826-835.2002 PMC127667

[40] von Andrian UH, Chambers JD, Berg EL, Michie SA, Brown DA, et al (1993) L-selectin mediates neutrophil rolling in inflamed venules through sialyl LewisX-dependent and-independent recognition pathways. Blood 82: 182–191. 7686786

[41] Palecanda A, Walcheck B, Bishop DK, Jutila MA (1992) Rapid activation-independent shedding of leukocyte L-selectin induced by cross-linking of the surface antigen. Eur J Immunol 22: 1279–1286. 1374339 10.1002/eji.1830220524

[42] Jutila MA, Rott L, Berg EL, Butcher EC (1989) Function and regulation of the neutrophil MEL-14 antigen in vivo: comparison with LFA-1 and MAC-1. J Immunol 143: 3318–3324. 2553811

[43] Berg M, James SP (1990) Human neutrophils release the Leu-8 lymph node homing receptor during cell activation. Blood 76: 2381–2388. 1701670

[44] Tavares NM, Araujo-Santos T, Afonso L, Nogueira PM, Lopes UG, et al (2014) Understanding the mechanisms controlling Leishmania amazonensis infection in vitro: the role of LTB4 derived from human neutrophils. J Infect Dis.210: 656–666. 10.1093/infdis/jiu158 24634497 PMC4111911

[45] Chang KP (1981) Leishmanicidal mechanisms of human polymorphonuclear phagocytes. Am J Trop Med Hyg 30: 322–333. 7235125 10.4269/ajtmh.1981.30.322

[46] Bisti S, Konidou G, Boelaert J, Lebastard M, Soteriadou K (2006) The prevention of the growth of Leishmania major progeny in BALB/c iron-loaded mice: a process coupled to increased oxidative burst, the amplitude and duration of which depend on initial parasite developmental stage and dose. Microbes Infect 8: 1464–1472. 16698303 10.1016/j.micinf.2006.01.014

[47] Lorne E, Zmijewski JW, Zhao X, Liu G, Tsuruta Y, et al (2008) Role of extracellular superoxide in neutrophil activation: interactions between xanthine oxidase and TLR4 induce proinflammatory cytokine production. Am J Physiol Cell Physiol 294: C985–993. 10.1152/ajpcell.00454.2007 18287332

[48] Mitra S, Abraham E (2006) Participation of superoxide in neutrophil activation and cytokine production. Biochim Biophys Acta 1762: 732–741. 16919916 10.1016/j.bbadis.2006.06.011

[49] Onnheim K, Bylund J, Boulay F, Dahlgren C, Forsman H (2008) Tumour necrosis factor (TNF)-alpha primes murine neutrophils when triggered via formyl peptide receptor-related sequence 2, the murine orthologue of human formyl peptide receptor-like 1, through a process involving the type I TNF receptor and subcellular granule mobilization. Immunology 125: 591–600. 10.1111/j.1365-2567.2008.02873.x 18710405 PMC2612554

[50] Ribeiro-Gomes FL, Moniz-de-Souza MC, Alexandre-Moreira MS, Dias WB, Lopes MF, et al (2007) Neutrophils activate macrophages for intracellular killing of Leishmania major through recruitment of TLR4 by neutrophil elastase. J Immunol 179: 3988–3994. 17785837 10.4049/jimmunol.179.6.3988

[51] Charmoy M, Megnekou R, Allenbach C, Zweifel C, Perez C, et al (2007) Leishmania major induces distinct neutrophil phenotypes in mice that are resistant or susceptible to infection. J Leukoc Biol 82: 288–299. 17449725 10.1189/jlb.0706440

[52] Savill JS, Wyllie AH, Henson JE, Walport MJ, Henson PM, et al (1989) Macrophage phagocytosis of aging neutrophils in inflammation. Programmed cell death in the neutrophil leads to its recognition by macrophages. J Clin Invest 83: 865–875. 2921324 10.1172/JCI113970 PMC303760

[53] Renshaw SA, Timmons SJ, Eaton V, Usher LR, Akil M, et al (2000) Inflammatory neutrophils retain susceptibility to apoptosis mediated via the Fas death receptor. J Leukoc Biol 67: 662–668. 10811006 10.1002/jlb.67.5.662

[54] Fadeel B, Ahlin A, Henter JI, Orrenius S, Hampton MB (1998) Involvement of caspases in neutrophil apoptosis: regulation by reactive oxygen species. Blood 92: 4808–4818. 9845548

[55] Simon HU, Haj-Yehia A, Levi-Schaffer F (2000) Role of reactive oxygen species (ROS) in apoptosis induction. Apoptosis 5: 415–418. 11256882 10.1023/a:1009616228304

[56] Sarkar A, Aga E, Bussmeyer U, Bhattacharyya A, Moller S, et al (2013) Infection of neutrophil granulocytes with Leishmania major activates ERK 1/2 and modulates multiple apoptotic pathways to inhibit apoptosis. Med Microbiol Immunol 202: 25–35. 10.1007/s00430-012-0246-1 22661217

[57] Mollinedo F, Janssen H, de la Iglesia-Vicente J, Villa-Pulgarin JA, Calafat J (2010) Selective fusion of azurophilic granules with Leishmania-containing phagosomes in human neutrophils. J Biol Chem 285: 34528–34536. 10.1074/jbc.M110.125302 20801889 PMC2966068

[58] van Zandbergen G, Klinger M, Mueller A, Dannenberg S, Gebert A, et al (2004) Cutting edge: neutrophil granulocyte serves as a vector for Leishmania entry into macrophages. J Immunol 173: 6521–6525. 15557140 10.4049/jimmunol.173.11.6521

[59] Ribeiro-Gomes FL, Peters NC, Debrabant A, Sacks DL (2012) Efficient capture of infected neutrophils by dendritic cells in the skin inhibits the early anti-leishmania response. PLoS Pathog 8: e1002536 10.1371/journal.ppat.1002536 22359507 PMC3280984

[60] Charmoy M, Auderset F, Allenbach C, Tacchini-Cottier F (2010) The prominent role of neutrophils during the initial phase of infection by Leishmania parasites. J Biomed Biotechnol 2010: 719361 10.1155/2010/719361 19884987 PMC2768872

[61] Bennouna S, Bliss SK, Curiel TJ, Denkers EY (2003) Cross-talk in the innate immune system: neutrophils instruct recruitment and activation of dendritic cells during microbial infection. J Immunol 171: 6052–6058. 14634118 10.4049/jimmunol.171.11.6052

[62] Megiovanni AM, Sanchez F, Robledo-Sarmiento M, Morel C, Gluckman JC, et al (2006) Polymorphonuclear neutrophils deliver activation signals and antigenic molecules to dendritic cells: a new link between leukocytes upstream of T lymphocytes. J Leukoc Biol 79: 977–988. 16501052 10.1189/jlb.0905526

