# Supplementary figures and images for: Exposure to Leishmania braziliensis Triggers Neutrophil Activation and Apoptosis
Source: PLoS Negl Trop Dis. 2015 Mar 10;9(3):e0003601. doi: 10.1371/journal.pntd.0003601 (PMC4354905; doi:10.1371/journal.pntd.0003601)

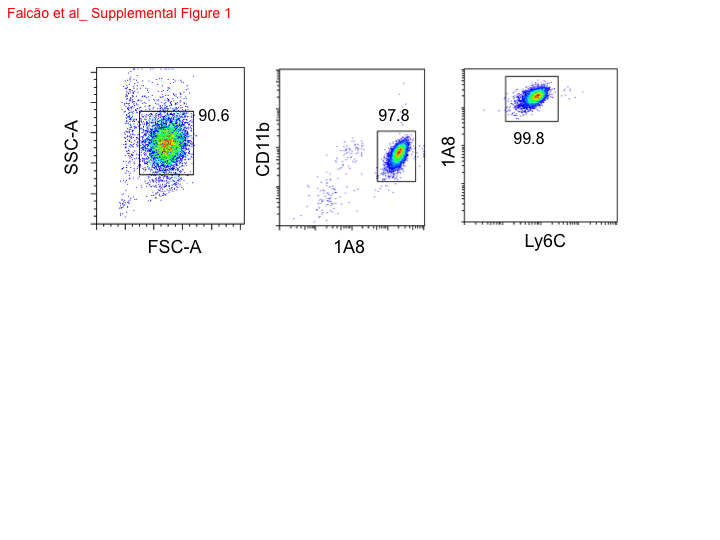

Supplement: S1 Fig — Inflammatory neutrophils were purified using MACS and the 1A8 (Ly6G) mAb. In this sample cells were first gated for size and granularity (SSC x FSC). The gated cells were further analyzed for expression of CD11b/1A8 or 1A8/Ly6C. (TIF) [file pntd.0003601.s001.tif]

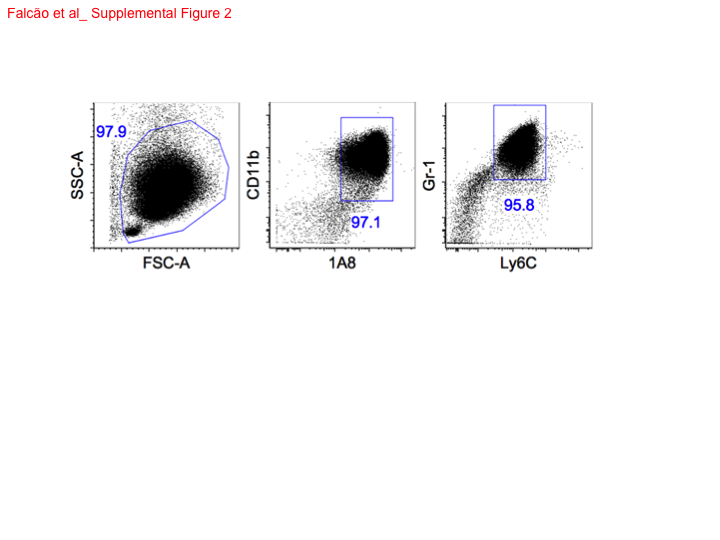

Supplement: S2 Fig — Bone marrow neutrophils were purified using MACS and the 1A8 (Ly6G) mAb. In this sample cells were first gated for size and granularity (SSC x FSC). The gated cells were further analyzed for expression of CD11b/1A8 or Gr-1/Ly6C. (TIF) [file pntd.0003601.s002.tif]

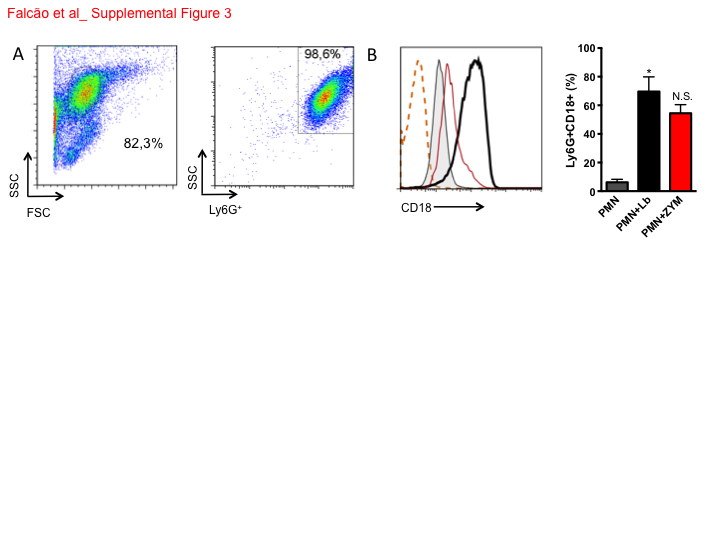

Supplement: S3 Fig — Inflammatory neutrophils were co-cultured with L. braziliensis or with Zymozan (SIGMA (100ug/ml). (A) Representative dot plots showing the gating strategy used to identify neutrophils (Ly6G) following exposure to L. braziliensis. Cells were stained with anti-CD18 (B) and were analyzed by FACS. Orange histograms: isotype control. Gray histogram: neutrophils cultured in medium only. Red histograms: neutrophils cultured with Zymozan. Black histograms, neutrophils exposed to L. braziliensis. Bar graphs represent the percentage of neutrophils positive for CD18. Data shown (mean ± SEM) are pooled from two independent repeats. *p<0.05 (One Way ANOVA). (TIF) [file pntd.0003601.s003.tif]
